# Cardiac stress imaging for the prediction of very long-term outcomes: Dobutamine stress echocardiography or dobutamine ^99m^Tc-sestamibi SPECT?

**DOI:** 10.1007/s12350-016-0521-4

**Published:** 2016-07-21

**Authors:** Hendrik J. Boiten, Ron T. van Domburg, Marcel L. Geleijnse, Roelf Valkema, Felix Zijlstra, Arend F. L. Schinkel

**Affiliations:** 1 Department of Cardiology, Thoraxcenter Room Ba304, Erasmus Medical Center, ‘s-Gravendijkwal 230, Rotterdam, 3015 CE The Netherlands; 2 Department of Nuclear Medicine, Thoraxcenter, Erasmus Medical Center, Rotterdam, The Netherlands

**Keywords:** DSE, myocardial perfusion imaging, SPECT, long-term prognostic value, cardiac events

## Abstract

**Background:**

Both dobutamine stress echocardiography (DSE) and myocardial perfusion imaging (MPI) using single-photon emission computed tomography (SPECT) are frequently used for cardiac risk stratification. The long-term relative prognostic value of these modalities has not been studied. Therefore, this study evaluated the long-term prognostic value of DSE compared to MPI in patients unable to perform exercise testing.

**Methods:**

This prospective, single center study included 301 patients (mean age 59 ± 12 years, 56% men) unable to perform exercise tests who underwent DSE and dobutamine stress ^99m^Tc-sestamibi MPI. End points during follow-up were all-cause mortality, cardiac mortality, and nonfatal myocardial infarction (MI). Univariable and multivariable Cox proportional hazards regression models were used to identify independent predictors of outcome. The probability of survival was calculated using the Kaplan-Meier method.

**Results:**

A total of 182 patients (60%) had an abnormal DSE and 198 (66%) patients had an abnormal MPI. The agreement between DSE and MPI was 82% (*κ* = 0.62). During a median follow-up of 14 years (range 5-18), 172 deaths (57%) occurred, of which 72 (24%) were due to cardiac causes. Nonfatal MI occurred in 46 patients (15%). The multivariable analysis demonstrated that an abnormal DSE was a significant predictor of cardiac mortality (HR 2.35, 95% CI [1.17-4.73]) and hard cardiac events (HR 2.11, 95% CI [1.25-3.57]). Also, an abnormal MPI result was a significant predictor of cardiac mortality (HR 3.03, 95% CI [1.33-6.95]) and hard cardiac events (HR 2.06, 95% CI [1.12-3.79]).

**Conclusions:**

DSE and MPI are comparable in predicting long-term cardiac mortality and hard cardiac events in patients unable to perform exercise testing.

**Electronic supplementary material:**

The online version of this article (doi:10.1007/s12350-016-0521-4) contains supplementary material, which is available to authorized users.

## Introduction

Coronary artery disease (CAD) remains a leading cause of morbidity and mortality worldwide. As such, non-invasive imaging techniques are important to diagnose and risk stratify patients with CAD.^1^ In patients who cannot exercise, due to conditions such as degenerative joint disease and peripheral vascular disease, pharmacologic stress testing is an appropriate alternative. Both dobutamine stress echocardiography (DSE) and stress myocardial perfusion imaging (MPI) are widely used for the evaluation of stress-induced myocardial ischemia, and provide significant prognostic information.^2^-^7^ Currently, information on the long-term relative prognostic value of DSE compared to MPI is lacking. Therefore, this study evaluated the long-term prognostic value of DSE compared to MPI in patients unable to perform exercise testing.

## Methods

### Study Population

The study population consisted of 354 consecutive patients. This study is a continuation of the previously reported study in which the same patient population was evaluated with a mean follow-up of 7.3 years.^3^ All patients underwent simultaneous dobutamine stress echocardiography and dobutamine stress ^99m^Tc-sestamibi SPECT for the evaluation of suspected or known CAD. Follow-up was successful in 351 (99%) patients. Fifty patients underwent early coronary revascularization <60 days after MPI and were excluded from analysis. This exclusion was based on previously published data indicating that referral to coronary revascularization in the first 60 days after testing tends to be based on the results of the test. Referral to revascularization >60 days after testing tends to be based on the worsening of the patient’s clinical status.^8^ All patients gave informed consent before testing and the local ethics committee approved the study protocol. Patients were enrolled between January 1991 and January 1995. The test in these patients with known or suspected CAD was requested for evaluation of ischemia. Before the stress test, a structured interview was achieved, including assessment of cardiac risk factors. Hypertension was defined as blood pressure ≥140/90 mmHg or the use of antihypertensive medication. Diabetes mellitus was defined as a fasting glucose level >140 mg/dL or use of insulin or oral hypoglycemic agents. Hypercholesterolemia was defined as a total cholesterol ≥6.4 mmol/L or treatment with lipid-lowering medication.

### Dobutamine Stress Testing Protocol

The dobutamine stress testing was performed using a standard protocol as described previously.^3^ Dobutamine was injected intravenously, starting at a dose of 10 µg/kg/min for 3 min and increasing by 10 µg/kg/min to a maximum dose of 40 µg/kg/min. If the test end point was not reached at a maximum dose of dobutamine, up to 1 mg of atropine was administered intravenously. During stress testing, blood pressure, heart rate, and electrocardiography were continuously monitored. Test endpoints were achievement of target heart rate (85% of maximum age and sex-predicted heart rate), horizontal or downsloping ST-segment depression of more than 2 mm, ST-segment elevation of more than 1 mm, severe angina, systolic blood pressure decrease >40 mmHg, blood pressure >240/110 mmHg, or clinically important cardiac arrhythmias. An intravenous beta-blocker was used to reverse the effects of dobutamine/atropine.

### ^99m^Tc-Sestamibi SPECT MPI

An intravenous dose of 370 MBq of ^99m^Tc-sestamibi was administered approximately 1 minute before the termination of the stress test. For resting studies, 370 MBq of ^99m^Tc-sestamibi was injected ≥24 hours after the stress study. Image acquisition was achieved with a Gammasonics single-head camera (Siemens, Iselin, NJ) without attenuation or scatter correction, using a low-energy all-purpose collimator. In all cases, transaxial tomograms were reconstructed; for each study, six short-axis and three sagittal long-axis slices were analyzed. To compare the rest and stress studies, each of the short-axis slides was divided into eight equal segments. The septal part of the two basal slices was excluded from analysis because this region corresponds to the fibrous portion of the interventricular septum and normally exhibits reduced uptake. The apical region was assessed from the three sagittal cross sections. A total of 47 segments per patient were analyzed. Data interpretation was performed visually and semiquantitatively. Circumferential profile analysis was used to assist visual analysis of images.

Stress and rest tomographic views were reviewed side-by-side by two experienced observers who were unaware of clinical data and echocardiograms, using a 5-point scoring system (1 = normal, 2 = slightly reduced, 3 = moderately reduced, 4 = severely reduced, 5 = absent uptake). Ischemia was defined as a perfusion defect on stress images that partially or completely resolved at rest in at least two contiguous segments. A region was classified as infarcted in the case of a perfusion defect on stress images in two or more contiguous segments, which persisted on rest images. An abnormal scan was considered in the presence of a fixed or reversible perfusion defect. To overcome misalignment between the myocardial perfusion data and the echocardiographic data, an identical six-segment model was used for both techniques. The 16 echocardiographic and 47 scintigraphic segments were regrouped into six major myocardial regions (anterior, septum anterior, septum inferior, inferoposterior, lateral, and apical).

### Dobutamine Stress Echocardiography

Two-dimensional echocardiograms were acquired at rest, during dobutamine stress testing, and during recovery. Two experienced observers, unaware of any other data, scored the echocardiograms using a standard 16-segment model. Regional wall motion and systolic wall thickening were scored on a 5-point scale (1 = normal, 2 = mild hypokinesia, 3 = severe hypokinesia, 4 = akinesia, 5 = dyskinesia). Ischemia was defined as new or worsened wall motion abnormalities during stress, indicated by an increase in wall motion score of ≥1 grade in one or more segments. Ischemia was not considered to be present when akinetic segments at rest became dyskinetic during stress. Dobutamine stress echocardiographic results were defined as abnormal if there was ischemia during stress or fixed wall motion abnormalities. Echocardiographic segments were grouped into six major segments for comparison with SPECT.^9^ Tests that were stopped prematurely in the absence of perfusion or wall motion abnormalities were considered negative.

### Follow-Up

Clinical outcome data were obtained in 2012 by contacting the patient, the patients’ general practitioners, civil registries, and reviewing hospital records. The date of the last review or consultation was used to calculate follow-up time. The endpoints were all-cause mortality, cardiac mortality, and nonfatal myocardial infarction (MI). Cardiac mortality was defined as death caused by MI, cardiac arrhythmias, refractory heart failure, or sudden death occurring without another explanation. The combined endpoint of cardiac mortality and nonfatal MI was considered as hard cardiac events. MI was defined according to the Joint European Society of Cardiology/American College of Cardiology Committee criteria.^10^ Diagnosis of an acute, evolving, or recent MI was fulfilled either by a typical rise and gradual fall (troponin) or more rapid rise and fall (creatinine kinase-MB) of biochemical markers of myocardial necrosis with at least one of the following criteria: ischemic symptoms, development of pathological Q waves on the electrocardiogram, electrocardiogram changes indicative of myocardial ischemia (ST-segment elevation or depression), or coronary artery intervention; or by pathological findings of an acute MI.

### Statistical Analysis

Values were expressed as means (±SD) or number, and compared using the Student *t*-test or Chi-squared test. Agreement between DSE and MPI was assessed by κ statistics. Univariable and multivariable Cox proportional hazard models were used to identify variables that were independently predictive of late cardiac events. The risk of a variable was expressed as hazard ratios with corresponding 95% confidence intervals. Clinical data, stress test variables, and non-invasive imaging data were incorporated into the analysis. The multivariable analysis was performed by first considering the clinical data, and next the combination of clinical and stress test variables. In the final models, the stress echocardiographic or ^99m^Tc-sestamibi SPECT data were added. The discriminative ability of the Cox regression models was determined by calculating the concordance (C)-index. Models are typically considered reasonable when the C-index >0.7.^11^ The probability of survival was calculated using the Kaplan-Meier method, and survival curves were compared using the log-rank test. *P*-values <.05 were considered statistically significant. All statistical analyses were performed using SPSS 22 (IBM Corp., Armonk, NY, USA).

## Results

### Patient Demographics and Stress Test Results

The clinical characteristics are presented in Table 1. The mean age of the 301 patients was 59 ± 12 years, and 56% were men. A total of 149 patients (50%) had a previous MI. During the dobutamine-atropine stress test, heart rate increased from a mean (±SD) of 69 ± 13 beats per minute to 136 ± 17 beats per minute (*P* < .0001), and systolic blood pressure increased from 136 ± 23 mm Hg to 147 ± 30 mm Hg (*P* < .001). Atropine, which was added in 129 patients (43%), was more frequently administered in patients taking beta-blockers (67% (84/125]) than in those who were not (26% (45/176], *P* < .0001). Side effects during stress testing included non-sustained ventricular tachycardia in 13 patients (4%), atrial fibrillation in 4 patients (1%), severe hypotension in 2 patients (0.7%), nausea in 15 patients (5%), and headache in 15 patients (5%). No patient experienced a MI or fatal complication. The test was terminated because of side effects in 17 patients (6%).

**Table 1:** Clinical characteristics

Variable (number and %)	Total	Cardiac mortality	No cardiac mortality	*P*-value	Cardiac events	No cardiac events	*P*-value
	*N* = 301	*N* = 72	*N* = 229		*N* = 118	*N* = 183	
Clinical data							
Age >70 years	55 (18)	22 (31)	33 (14)	.65	23 (19)	32 (17)	.38
Male gender	168 (56)	50 (69)	118 (52)	.01	79 (67)	89 (49)	.002
Previous MI	149 (50)	51 (71)	98 (43)	<.001	79 (34)	70 (38)	<.001
Hypertension	132 (44)	34 (47)	98 (43)	.49	54 (46)	78 (43)	.45
Hypercholesterolemia	76 (25)	23 (32)	53 (23)	.21	34 (29)	42 (23)	.27
Smoking	79 (26)	26 (36)	53 (23)	.03	34 (29)	45 (25)	.42
Heart failure	57 (19)	26 (36)	31 (14)	<.001	30 (25)	27 (15)	.003
Diabetes mellitus	43 (14)	14 (19)	29 (13)	.16	19 (16)	24 (13)	.55
Stress test results							
Typical angina	72 (24)	16 (22)	56 (24)	.69	33 (28)	39 (21)	.21
ST-segment changes	85 (28)	31 (43)	54 (24)	.001	47 (21)	38 (21)	.001
Abnormal DSE	182 (60)	59 (82)	123 (54)	<.001	92 (78)	90 (49)	<.001
Abnormal MPI	198 (66)	62 (86)	136 (59)	<.001	97 (82)	101 (55)	<.001

*MI*, myocardial infarction; *DSE* dobutamine stress echocardiography; *MPI* myocardial perfusion imaging

### Imaging Results and Follow-Up

Abnormal DSE was detected in 182 (60%) patients, whereas 198 (66%) patients had an abnormal MPI. The agreement between DSE and MPI was 82% (*κ* = 0.62). When considered the 152 patients without previous MI the agreement between DSE and MPI was 81% (*κ* = 0.60). The agreement between DSE and MPI was 86% (*κ* = 0.36) when only ischemia was considered. Normal DSE and abnormal MPI were observed in 35 patients, whereas 19 patients had a normal MPI and an abnormal DSE. During a median follow-up of 14 years (range 5-18), 172 deaths (57%) occurred, of which 72 (24%) were due to cardiac causes. Nonfatal MI occurred in 46 patients (15%).

### Clinical Data and Outcome

Univariable predictors of both endpoints (cardiac mortality and hard cardiac events) were age, male gender, previous MI, heart failure, and ST-segment changes (Table 1). When analyzed as a dichotomous variable age was not a significant predictor. Smoking was also a significant predictor of cardiac mortality. Both an abnormal DSE and an abnormal MPI were strongly associated with both endpoints.

### Survival Analysis and Predictors of Long-Term Outcome

Annualized event rates for cardiac mortality for patients with normal DSE was significantly lower (0.8%) than for those with abnormal DSE (2.8%, *P* < .001). According to hard cardiac events, annualized event rates for normal DSE were 1.5% compared to 3.8% in abnormal DSE (*P* < .001). Equally, the annualized event rates for cardiac mortality for patients with normal MPI was significantly lower than for those with abnormal MPI (0.6% vs 2.8%, *P* < .001). The annualized rates for hard cardiac events were 1.3% in normal MPI and 3.7% in abnormal MPI (*P* < .001).

**Figure 1:**
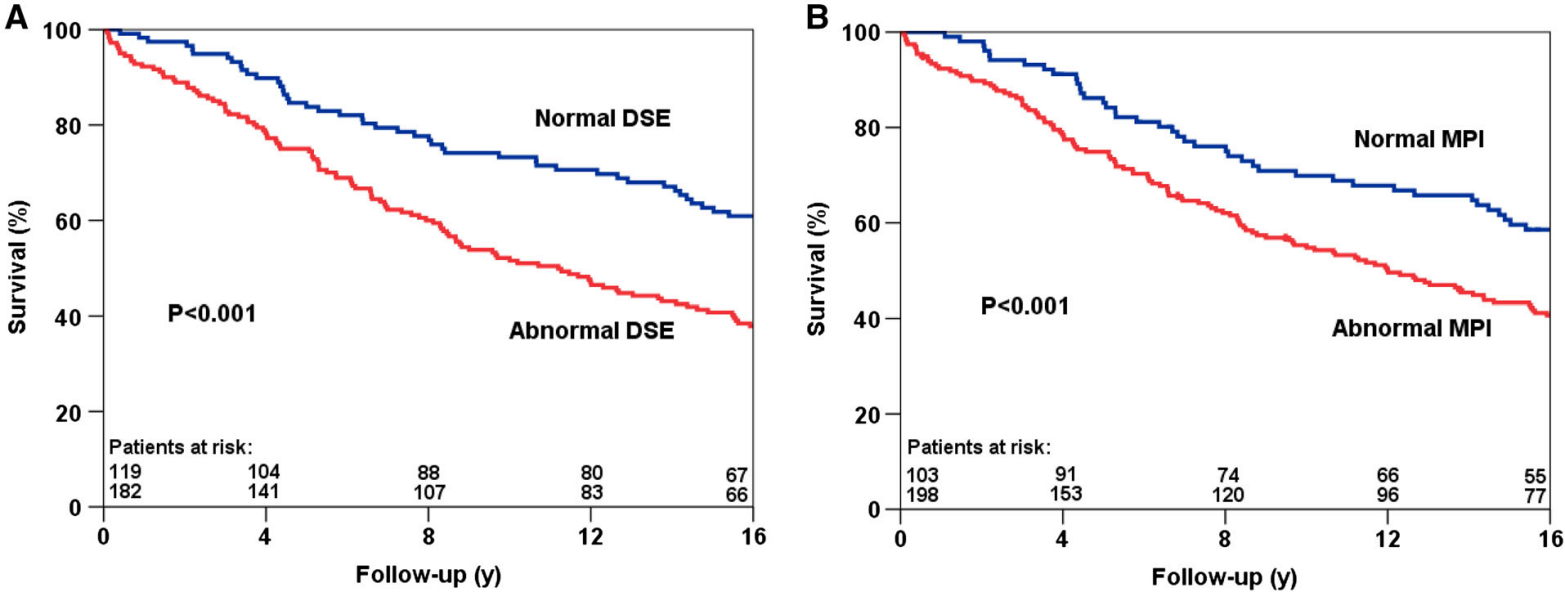
Kaplan-Meier survival curves for all-cause mortality using DSE (**A**) and MPI (**B**). *y* years, *MPI* myocardial perfusion imaging, *DSE* dobutamine stress echocardiography

**Figure 2:**
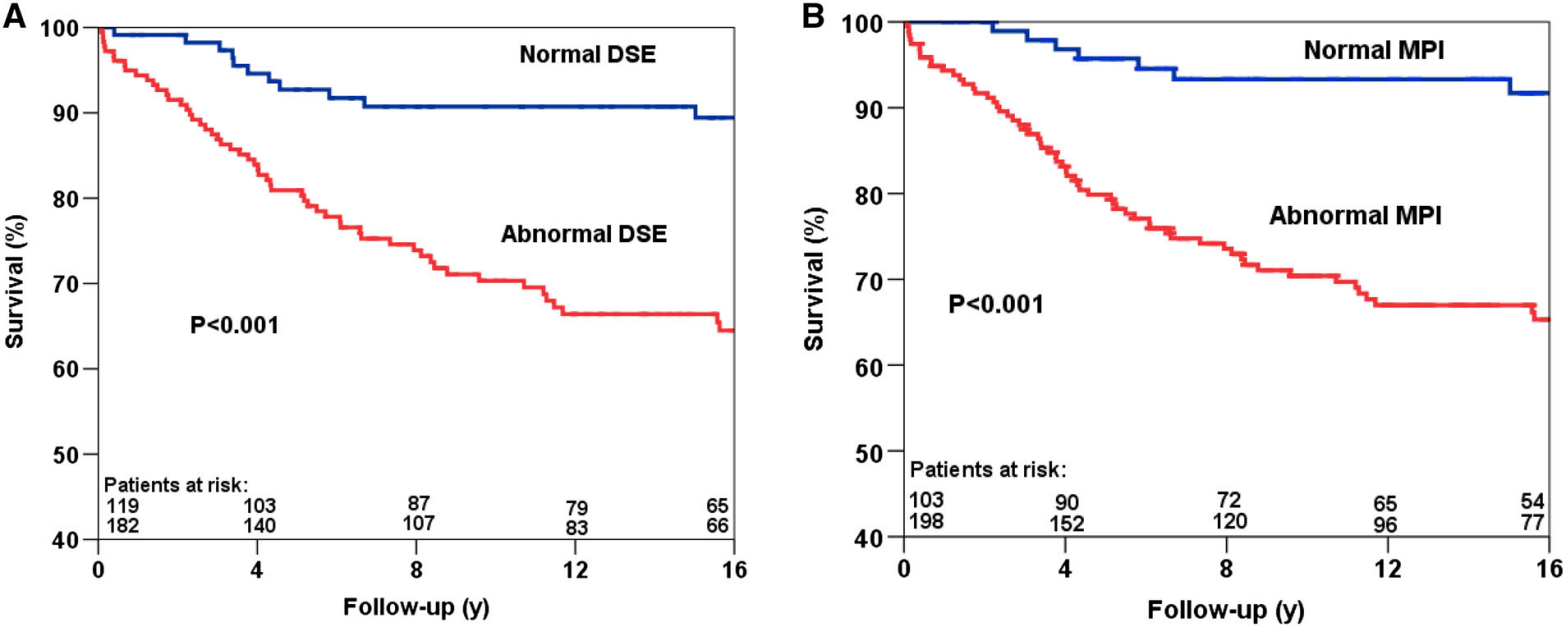
Kaplan-Meier survival curves for cardiac mortality using DSE (**A**) and MPI (**B**). *y*, years; *MPI*, myocardial perfusion imaging; *DSE*, dobutamine stress echocardiography

**Figure 3:**
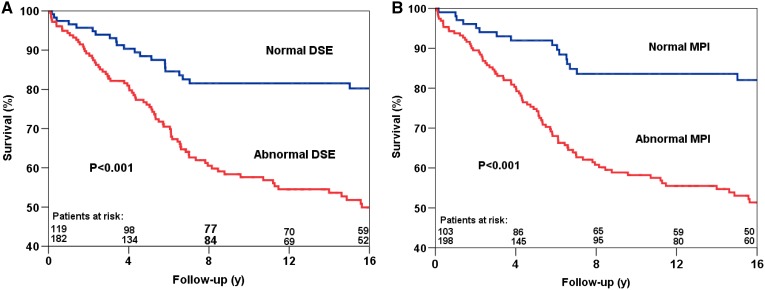
Kaplan-Meier survival curves for hard cardiac events using DSE (**A**) and MPI (**B**). *y*, years; *MPI*, myocardial perfusion imaging; *DSE*, dobutamine stress echocardiography

Kaplan-Meier survival curves are presented in Figures 1, 2, and 3. The survival curves show that a normal DSE and a normal MPI were associated with relatively low risk for all-cause mortality, cardiac mortality, and hard cardiac events. Conversely, patients with an abnormal test result had a significantly increased risk of all-cause mortality (Figure 1), cardiac mortality (Figure 2) and hard cardiac events (Figure 3???). The survival curves continued to diverge during the long-term follow-up period, which indicated a maintained prognostic value of both imaging modalities.

Multivariable analysis demonstrated that age, previous MI, smoking, and heart failure were independent predictors of cardiac mortality (Table 2). Previous MI and heart failure were independent predictors of hard cardiac events (Table 2). Age was of borderline significance. A multivariable model also revealed that both an abnormal DSE and an abnormal MPI had an incremental prognostic value over clinical variables and stress test parameters. For the prediction of cardiac mortality, the C-index for clinical and clinical and stress test data was 0.73 and 0.76, respectively. The C-index was 0.76 when DSE or MPI was added. For the prediction of hard cardiac events, the C-index for clinical and clinical and stress test data was 0.70 and 0.71, respectively and 0.71 when DSE or MPI were added.

**Table 2:** Multivariable predictors of cardiac mortality and hard cardiac events

Variable	Cardiac mortality				Hard cardiac events			
	Clinical data	Clinical data + stress test	Clinical data + stress test + SPECT	Clinical data + stress test + ECHO	Clinical data	Clinical data + stress test	Clinical data + stress test + SPECT	Clinical data + stress test + ECHO
Clinical data								
Age*	1.04 (1.01–1.06)	1.04 (1.02–1.06)	1.04 (1.02–1.07)	1.04 (1.02–1.06)	*P* = .05	1.02 (1.01–1.04)	1.02 (1.00–1.04)	1.02 (1.00–1.04)
Men	*P* = .25	*P* = .36	*P* = .36	*P* = .30	*P* = .10	*P* = .08	*P* = .15	*P* = .09
Previous MI**	2.32 (1.34–4.01)	2.16 (1.24–3.75)	*P* = .09	*P* = .06	2.25 (1.47–3.45)	2.06 (1.33–3.17)	1.69 (1.06–2.68)	*P* = .05
Hypertension	*P* = .29	*P* = .29	*P* = .80	*P* = .90	*P* = .37	*P* = .18	*P* = .24	*P* = .73
Hypercholesterolemia	*P* = .23	*P* = .23	*P* = .14	*P* = .17	*P* = .94	*P* = .72	*P* = .73	*P* = .48
Smoking	1.88 (1.14–3.10)	1.78 (1.08–2.94)	1.78 (1.08–2.95)	1.89 (1.14–3.12)	*P* = .67	*P* = .54	*P* = .82	*P* = .42
Heart failure	2.52 (1.52–4.18)	2.39 (1.43–3.97)	2.29 (1.38–3.82)	2.31 (1.38–3.88)	1.77 (1.14–2.76)	1.63 (1.04–2.54)	*P* = .08	1.62 (1.03–2.55)
Diabetes mellitus	*P* = .28	*P* = .41	*P* = .24	*P* = .27	*P* = .87	*P* = .93	*P* = .86	*P* = .71
Stress test variables								
Typical angina	–	*P* = .25	*P* = .39	*P* = .33	–	*P* = .83	*P* = .82	*P* = .54
ST–segment changes	–	1.93 (1.18–3.14)	1.69 (1.03–2.77)	1.78 (1.08–2.93)	–	1.85 (1.23–2.77)	1.67 (1.11–2.52)	1.61 (1.05–2.45)
Abnormal MPI	–	–	3.03 (1.33–6.95)	–	–	–	2.06 (1.12–3.79)	–
Abnormal DSE	–	–	–	2.35 (1.17–4.73)	–	–	–	2.11 (1.25–3.57)
C-index	.73	.76	.76	.76	.70	.71	.71	.71

Values are expressed as Cox proportional hazard ratio (HR) and 95% confidence interval (CI)

*MI*, myocardial infarction

* Per 10% increase

## Discussion

The present study is the first to assess the prognostic value of DSE compared to MPI for long-term outcome. During a median follow-up of 14 years, 172 deaths occurred, of which 72 were due to cardiac causes. Nonfatal MI occurred in 46 patients. The survival curves continued to diverge during the follow-up period, indicating a maintained prognostic value of both DSE and MPI. Both patients with a normal DSE and a normal MPI had a relatively favorable long-term prognosis in contrast to patients with an abnormal imaging result. A multivariable model revealed that both an abnormal DSE and an abnormal MPI had an incremental prognostic value over clinical variables and stress test parameters. Ischemia on DSE but not ischemia on MPI was predictive of outcome. Both modalities were comparable in identifying patients with low or high risk of cardiac events during long-term follow-up.

Non-invasive imaging techniques are central in diagnosing patients with known of suspected CAD. Both DSE and MPI play important roles in this regard, in particular in patients with an intermediate likelihood of CAD,^12^ and show similar accuracy in the diagnosis of CAD.^5^,^6^ Data on the long-term prognostic implications of these imaging techniques are lacking.^13^

A few previous studies have compared the prognostic value between DSE and MPI with a mean follow-up time of less than 8 years.^2^,^3^,^5^-^7^,^14^,^15^ Geleijnse et al^2^ studied 220 patients with chest pain undergoing DSE and simultaneous ^99m^Tc-sestamibi SPECT. 24 patients experienced hard cardiac events. During a follow-up of 31 ± 15 months, event rates after both stress modalities were similar. They concluded that DSE and MPI provided comparable prognostic information. Olmos et al^14^ reported a study of 248 patients who underwent exercise echocardiography and thallium-201 SPECT. During a mean follow-up of 3.7 ± 2.0 years, 8 nonfatal infarctions and 7 cardiac deaths occurred. They reported that exercise echocardiography and thallium-201 SPECT provide comparable prognostic information for cardiac death and cardiac events. In 146 patients with previous MI, Acampa et al^15^ compared the prognostic value of DSE and SPECT MPI. During a mean follow-up of 44 ± 19 months, 20 cardiac events occurred. Ischemia at MPI, but not at echocardiography, was a significant predictor of cardiac events. Previously, we reported the 7-year follow-up in these 301 patients. In that study, patients with normal DSE and MPI maintained a relatively low event rate compared to patients with an abnormal DSE and MPI. Compared to these previous studies, our study included 301 patients who were followed for a median time of 14 years. The present study extends the observations from these previous studies and demonstrates that the long-term prognostic value of DSE is similar to that of stress MPI.

A meta-analysis reported higher annualized cardiac events for patients with normal pharmacological SPECT (1.78%) compared to patients with normal exercise SPECT (0.65%).^16^ Several reasons have been attributed for these difference; patients who underwent pharmacological stress SPECT are older, have more co-morbidities and has an increased number of risk factors for CAD. In the current study, we found an annualized hard cardiac event rate of 1.3% for normal dobutamine stress MPI. From a clinical perspective, patients unable to perform exercise tests who have a normal MPI have a good prognosis and could be spared invasive evaluation of the coronary arteries. Based on the current findings, both a normal DSE and a normal stress MPI identified low- and high-risk patient groups. As a consequence, both stress modalities could be used interchangeably in identifying low-risk patient group.

Both DSE and MPI have developed rapidly over the past years and have emerged as a valuable tool for diagnosis and prognosis of CAD. These imaging methods have inherent differences: SPECT probes myocardial hypoperfusion, whereas DSE probes systolic dysfunction.^13^ According to the ischemic cascade, a series of biochemical reactions that occurs after inadequate myocardial blood supply, perfusion abnormalities precede systolic dysfunction.^13^ This may influence the sensitivity and specificity of MPI and DSE for the evaluation of myocardial ischemia. DSE is less sensitive in detecting CAD for mild disease, but more specific for the overall patient group. Stress SPECT has a higher diagnostic accuracy in patients with multivessel CAD.^17^ A recent multicenter study 18 compared commonly used imaging techniques and found that both MPI and stress echocardiography had good diagnostic accuracy for CAD (area under the curve 0.74 and 0.70, respectively). Our findings show that there are comparable implications in risk-stratifying patients, using either modality. Which of these modalities is most suitable still depends on the patients clinical status, availability, and local expertise and costs.

This study has some limitations. First, the prognostic value of gated SPECT was not examined, due to the fact that at the time of data collection gated SPECT was not routinely performed in our center. As a result left ventricular ejection fraction (LVEF) was not available. Also, LVEF was not routinely examined during DSE. Information about LVEF could have improved the current analysis. Second, attenuation or scatter correction during stress SPECT was not routinely performed. Previous studies have shown that attenuation correction contribute to optimize further risk stratification.^19^,^20^ Third, the patient population was relatively small. This could have influenced the results.

## New Knowledge Gained

Dobutamine stress echocardiography and dobutamine stress _99m_Tc-sestamibi SPECT are comparable in predicting long-term (>14 years) cardiac mortality and hard cardiac events in high-risk patients (patients unable to perform exercise testing). From a clinical view, both techniques can be used interchangeably to classify patients as low or high risk of cardiac events.

## Conclusions

In this study DSE and MPI provide comparable prognostic information for the prediction of cardiac mortality and hard (cardiac mortality and nonfatal MI) cardiac events in patients unable to perform exercise testing. Both techniques can be used interchangeably to classify patients as low or high risk of cardiac events.

## Electronic supplementary material

Below is the link to the electronic supplementary material.

(PPTX 404 kb)

## Abbreviations

CAD: Coronary artery disease
C-statistic: Concordance statistic
DSE: Dobutamine stress echocardiography
ECG: Electrocardiography
LVEF: Left ventricular ejection fraction
MBq: Megabecquerel
MI: Myocardial infarction
MPI: Myocardial perfusion imaging
SPECT: Single-photon emission computed tomography
^99m^Tc: Technetium-99m
