# Supplementary material for: Cardiac stress imaging for the prediction of very long-term outcomes: Dobutamine stress echocardiography or dobutamine 99mTc-sestamibi SPECT?
Source: J Nucl Cardiol. 2016 Jul 21;25(2):471–9. doi: 10.1007/s12350-016-0521-4 (PMC5869882; doi:10.1007/s12350-016-0521-4)
Supplement: Supplementary file 1 — (PPTX 404 kb) [file 12350_2016_521_MOESM1_ESM.pptx]

## Slide 1
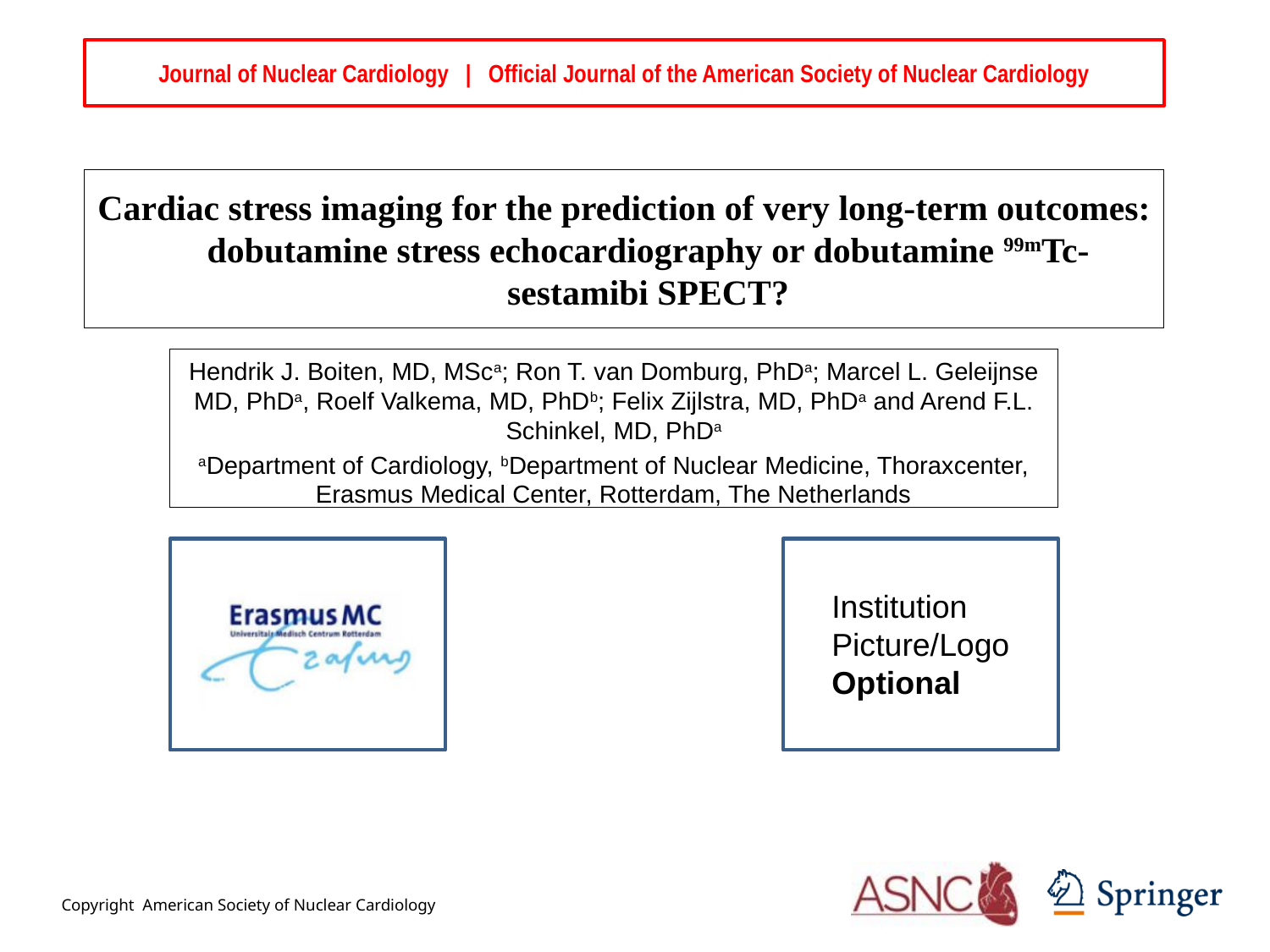

Journal of Nuclear Cardiology | Official Journal of the American Society of Nuclear Cardiology
# Cardiac stress imaging for the prediction of very long-term outcomes: dobutamine stress echocardiography or dobutamine 99mTc-sestamibi SPECT?
Hendrik J. Boiten, MD, MSca; Ron T. van Domburg, PhDa; Marcel L. Geleijnse MD, PhDa, Roelf Valkema, MD, PhDb; Felix Zijlstra, MD, PhDa and Arend F.L. Schinkel, MD, PhDa
aDepartment of Cardiology, bDepartment of Nuclear Medicine, Thoraxcenter, Erasmus Medical Center, Rotterdam, The Netherlands
Head shot of author
required
Institution
Picture/Logo
Optional
Copyright American Society of Nuclear Cardiology

## Slide 2
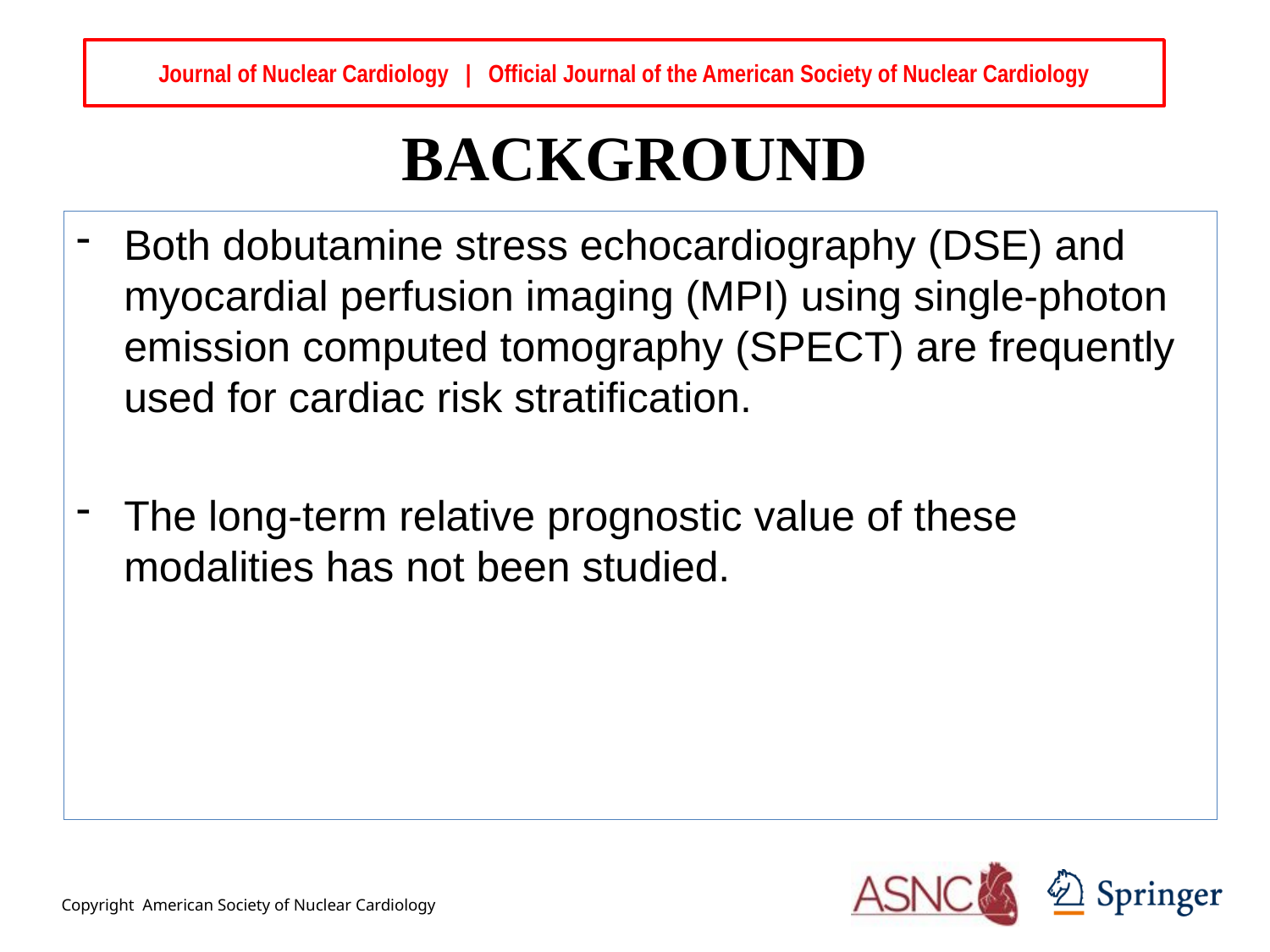

Journal of Nuclear Cardiology | Official Journal of the American Society of Nuclear Cardiology
# BACKGROUND
Both dobutamine stress echocardiography (DSE) and myocardial perfusion imaging (MPI) using single-photon emission computed tomography (SPECT) are frequently used for cardiac risk stratification.
The long-term relative prognostic value of these modalities has not been studied.
Copyright American Society of Nuclear Cardiology

## Slide 3
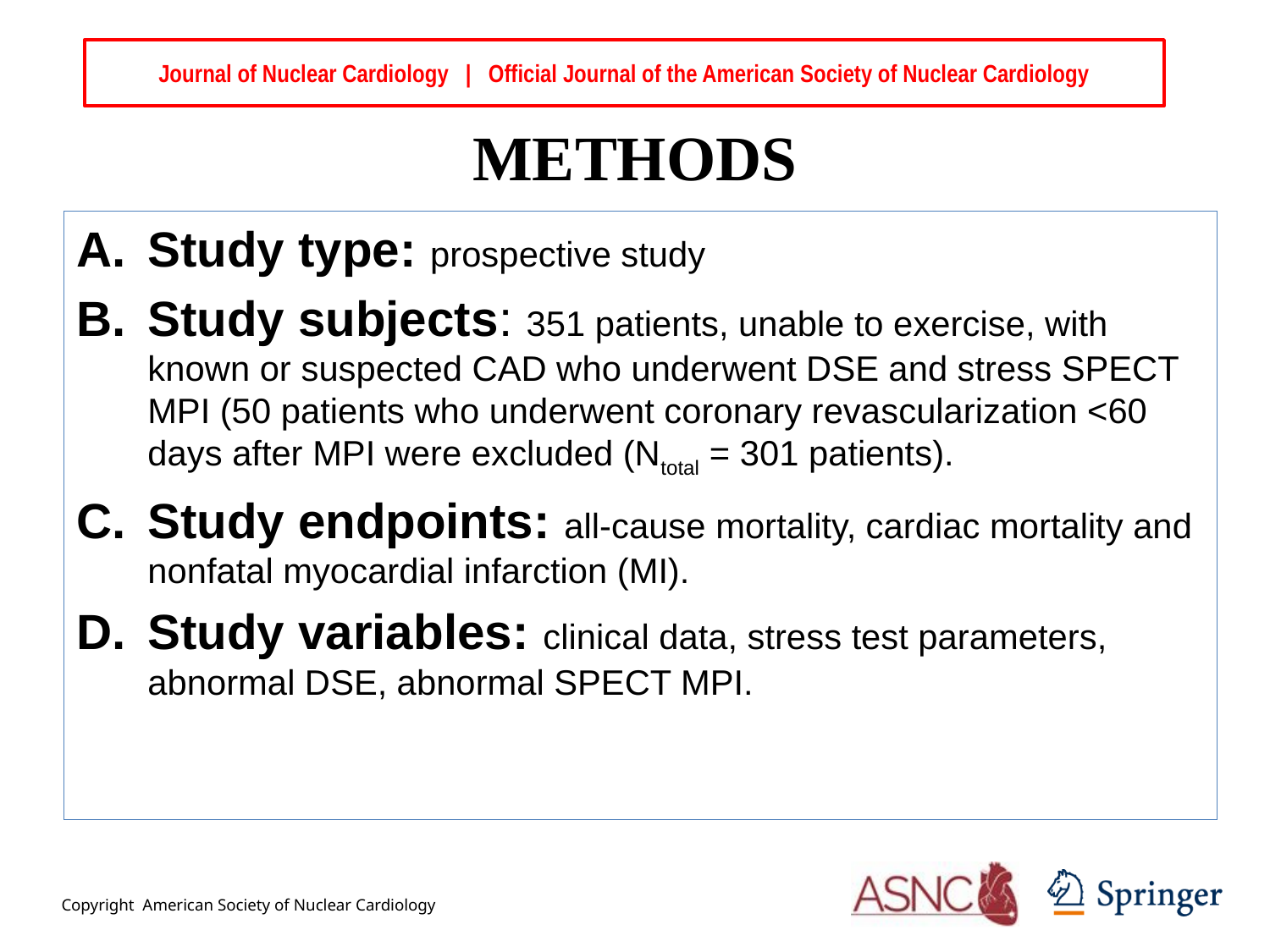

Journal of Nuclear Cardiology | Official Journal of the American Society of Nuclear Cardiology
# METHODS
Study type: prospective study
Study subjects: 351 patients, unable to exercise, with known or suspected CAD who underwent DSE and stress SPECT MPI (50 patients who underwent coronary revascularization <60 days after MPI were excluded (Ntotal = 301 patients).
Study endpoints: all-cause mortality, cardiac mortality and nonfatal myocardial infarction (MI).
Study variables: clinical data, stress test parameters, abnormal DSE, abnormal SPECT MPI.
Copyright American Society of Nuclear Cardiology

## Slide 4
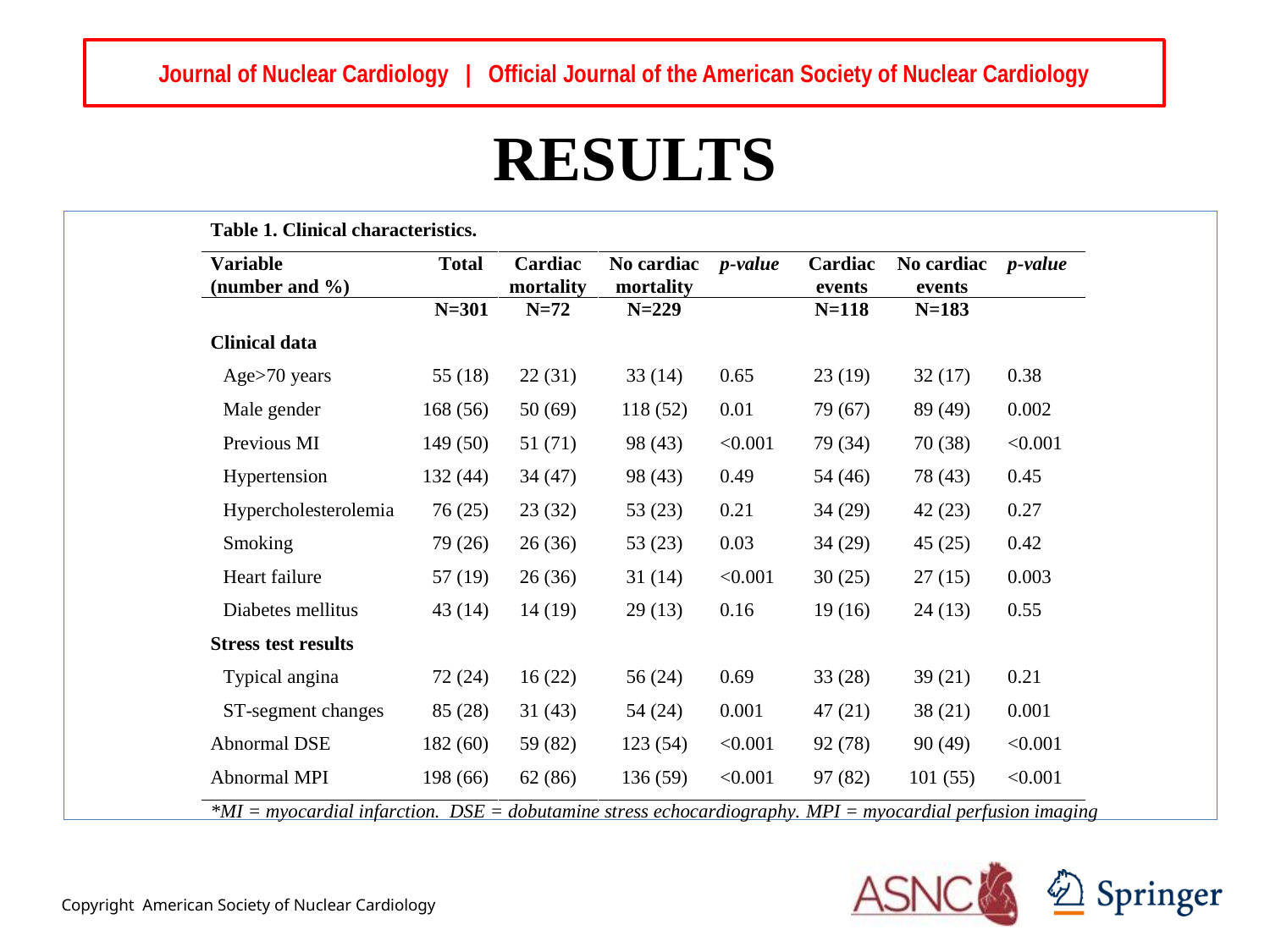

Journal of Nuclear Cardiology | Official Journal of the American Society of Nuclear Cardiology
# RESULTS
Copyright American Society of Nuclear Cardiology

## Slide 5
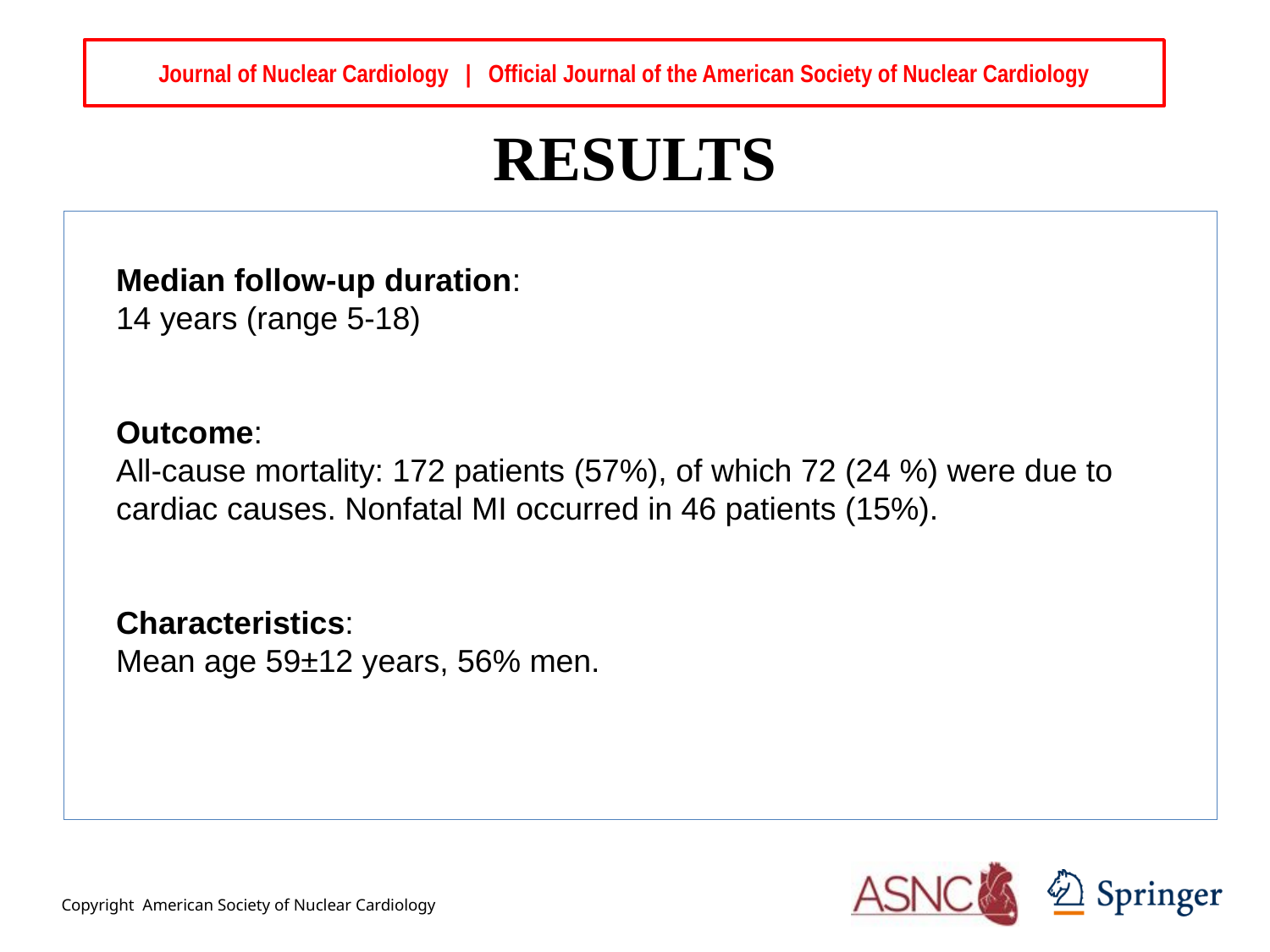

Journal of Nuclear Cardiology | Official Journal of the American Society of Nuclear Cardiology
# RESULTS
Median follow-up duration:
14 years (range 5-18)
Outcome:
All-cause mortality: 172 patients (57%), of which 72 (24 %) were due to cardiac causes. Nonfatal MI occurred in 46 patients (15%).
Characteristics:
Mean age 59±12 years, 56% men.
Copyright American Society of Nuclear Cardiology

## Slide 6
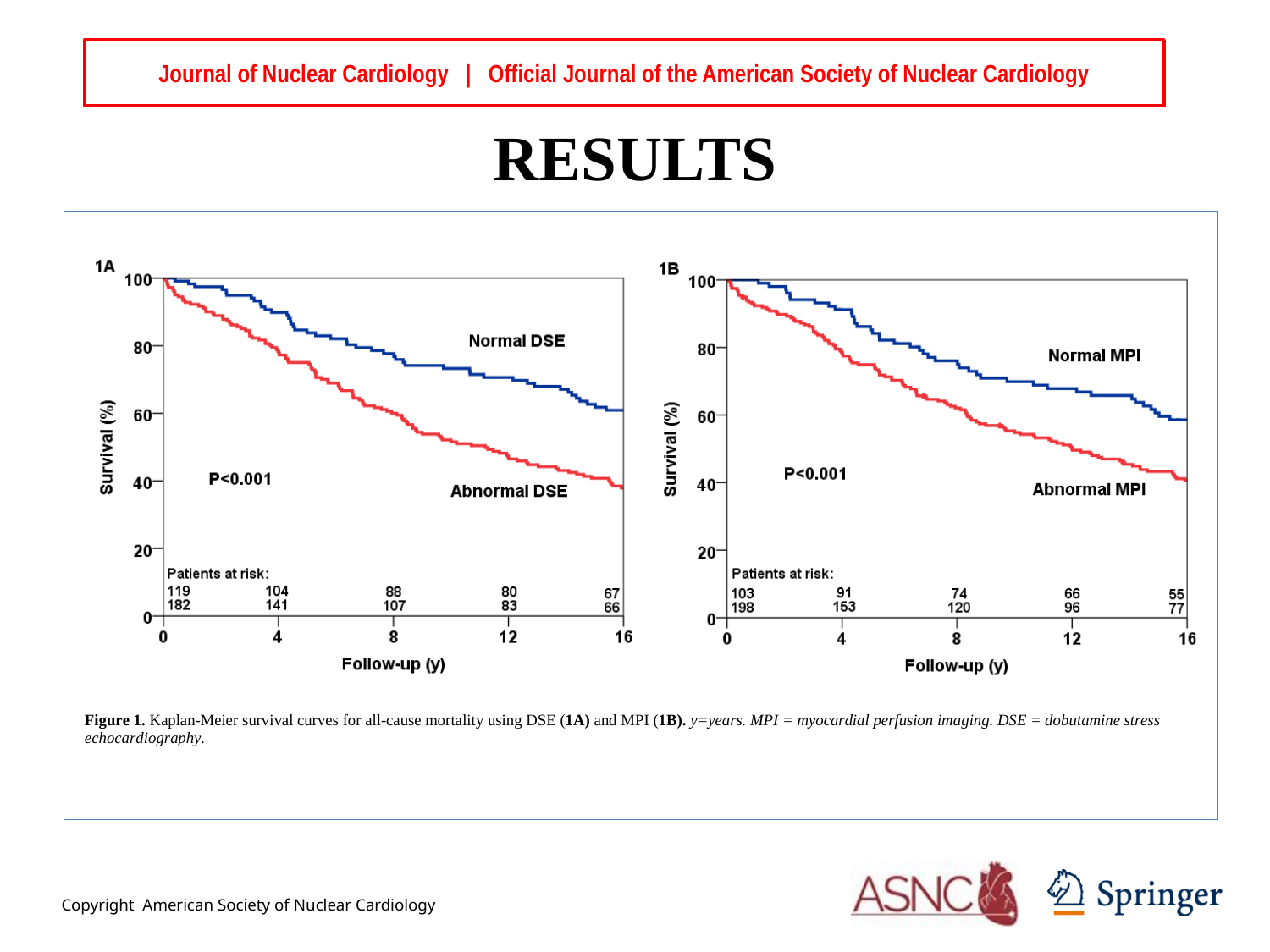

Journal of Nuclear Cardiology | Official Journal of the American Society of Nuclear Cardiology
# RESULTS
Copyright American Society of Nuclear Cardiology

## Slide 7
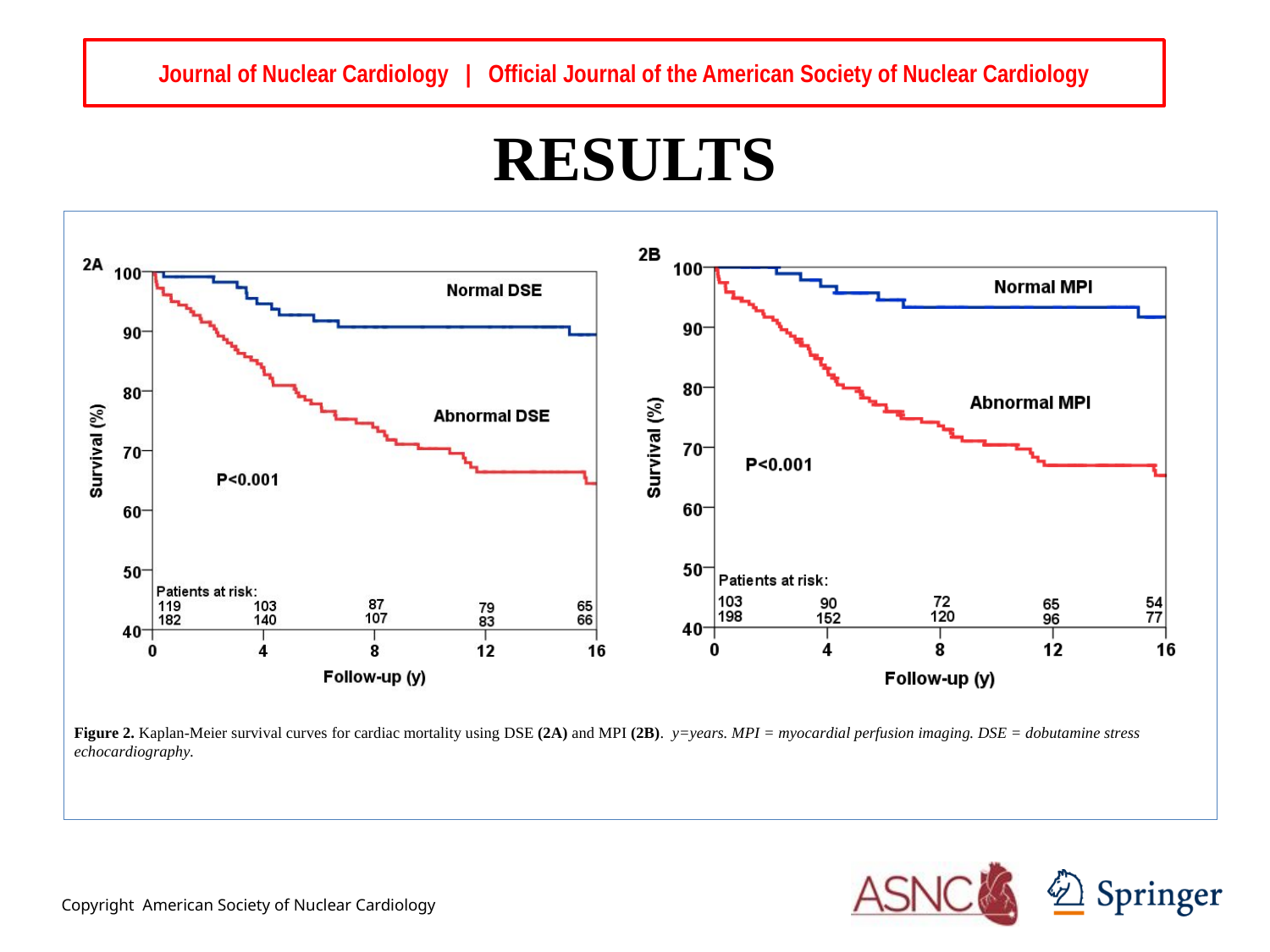

Journal of Nuclear Cardiology | Official Journal of the American Society of Nuclear Cardiology
# RESULTS
Copyright American Society of Nuclear Cardiology

## Slide 8
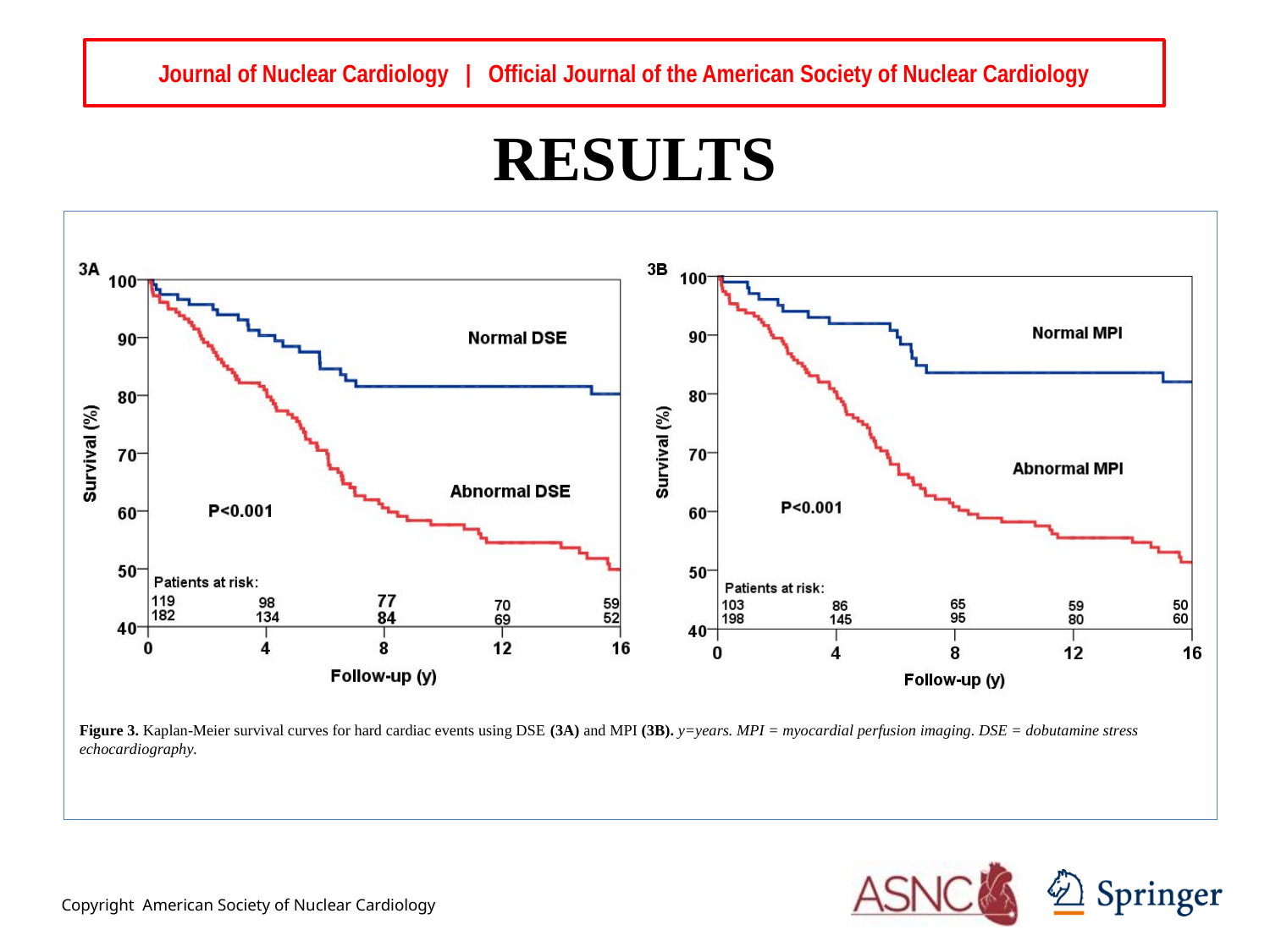

Journal of Nuclear Cardiology | Official Journal of the American Society of Nuclear Cardiology
# RESULTS
Copyright American Society of Nuclear Cardiology

## Slide 9
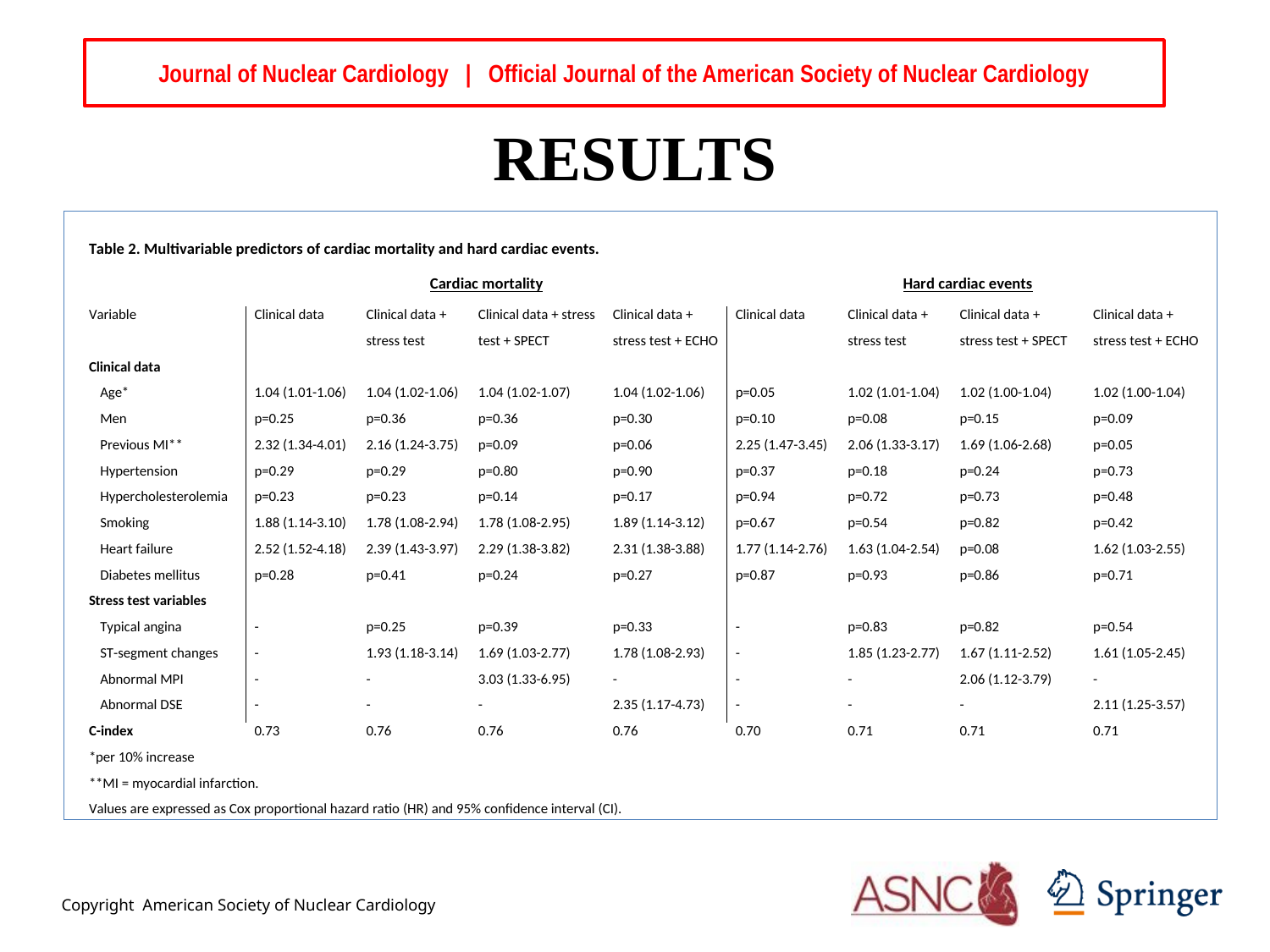

Journal of Nuclear Cardiology | Official Journal of the American Society of Nuclear Cardiology
# RESULTS
Copyright American Society of Nuclear Cardiology

## Slide 10
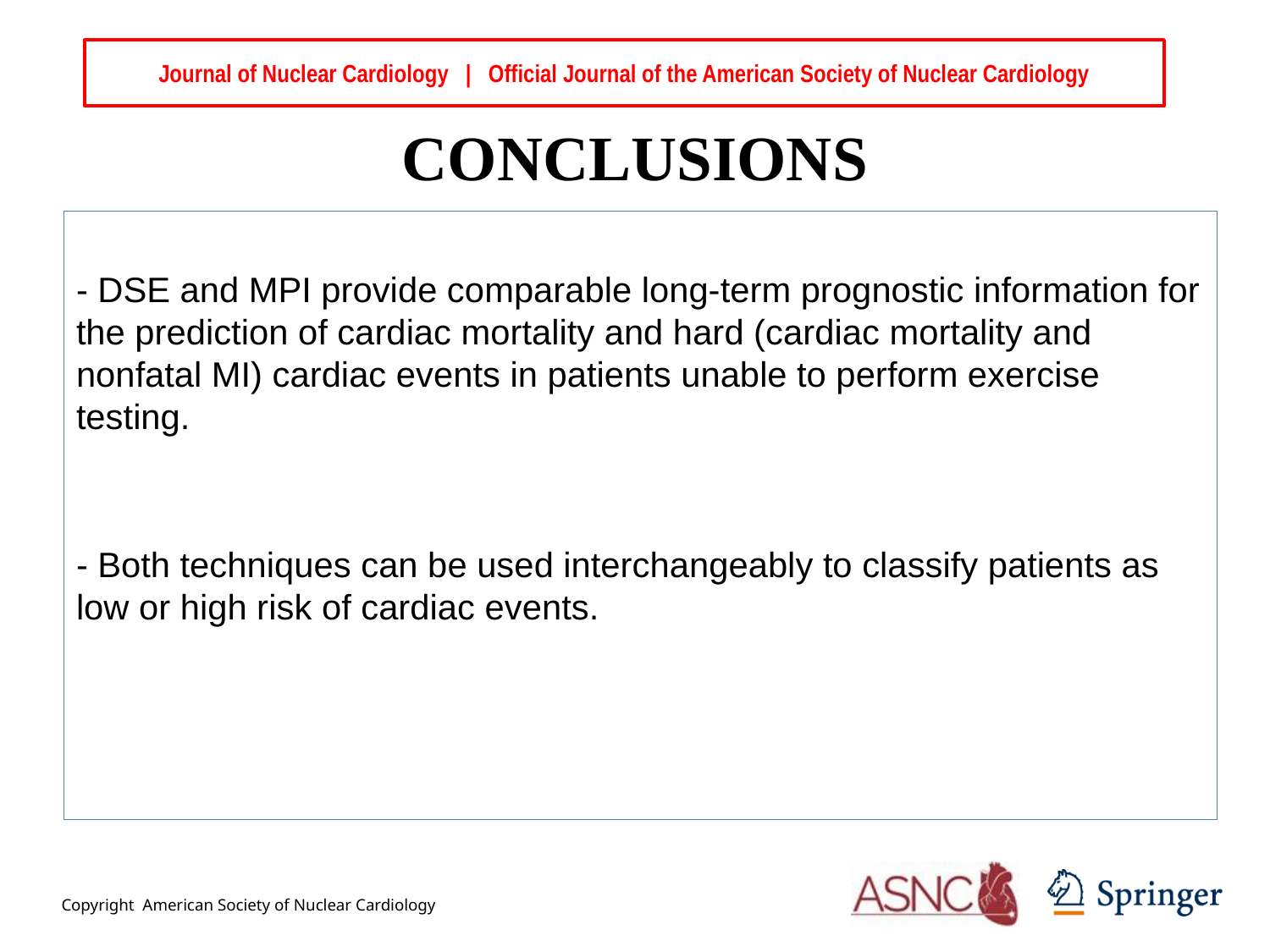

Journal of Nuclear Cardiology | Official Journal of the American Society of Nuclear Cardiology
# CONCLUSIONS
- DSE and MPI provide comparable long-term prognostic information for the prediction of cardiac mortality and hard (cardiac mortality and nonfatal MI) cardiac events in patients unable to perform exercise testing.
- Both techniques can be used interchangeably to classify patients as low or high risk of cardiac events.
Copyright American Society of Nuclear Cardiology
